# Immediate Articaine Allergy in Pediatric Dentistry: A Case Report of IgE-Mediated Hypersensitivity

**DOI:** 10.7759/cureus.94232

**Published:** 2025-10-09

**Authors:** Nabil Tiresse, Sara Bazia, Mohamed Baaouiss, Hanane Elouazzani

**Affiliations:** 1 Department of Pneumology, Mohammed V Military Training Hospital, Rabat, MAR

**Keywords:** allergy test, articaine hydrochloride, ige-mediated allergy, immediate hypersensitivity, local anesthetics

## Abstract

Local anesthetics (LAs) are widely used in dentistry and minor surgical procedures in children and are classified into two groups: esters and amides. Articaine, an amide derivative, is among the most effective and well-tolerated agents for dental procedures. Commercially available formulations are often combined with epinephrine. With the widespread use of these agents, reports of hypersensitivity reactions have emerged. We describe the case of an 11-year-old girl with asthma who developed immediate hypersensitivity to articaine during a dental procedure and was subsequently referred for allergological evaluation prior to additional dental care. Allergy to articaine, as with other LAs, is estimated to occur in approximately 1% of suspected cases. Most hypersensitivity reactions are attributed to vasovagal mechanisms related to anxiety, direct pharmacologic effects of epinephrine, or allergy to preservatives contained in anesthetic preparations. Allergological evaluation, guided by a thorough clinical history, involves performing skin prick tests, intradermal tests, and drug provocation tests based on the results of initial skin testing, in accordance with current guidelines. In our patient, the provocation test confirmed an IgE-mediated allergy to the LA, evidenced by cutaneous reactions and bronchospasm, along with elevated serum tryptase, highlighting the severity of the reaction. This case adds to the limited literature on LA allergy and underscores the importance for dental practitioners to recognize such reactions, acquire appropriate management skills, and ensure the availability of emergency treatments to address potential hypersensitivity events.

## Introduction

Local anesthetics (LAs) are commonly used in dentistry and minor surgical procedures [1]. Their widespread use has inevitably been associated with occasional adverse reactions, including hypersensitivity reactions [2]. Most reported reactions during LA administration are due to mechanisms unrelated to true IgE-mediated allergy. In children, true allergic reactions to LAs are exceedingly rare, with an estimated prevalence of approximately 1% [3]. We report the case of an 11-year-old girl who experienced an immediate hypersensitivity reaction to articaine, confirmed through allergological testing. This represents one of the few reported cases of IgE-mediated allergy to articaine to date.

## Case presentation

**Figure 1:**
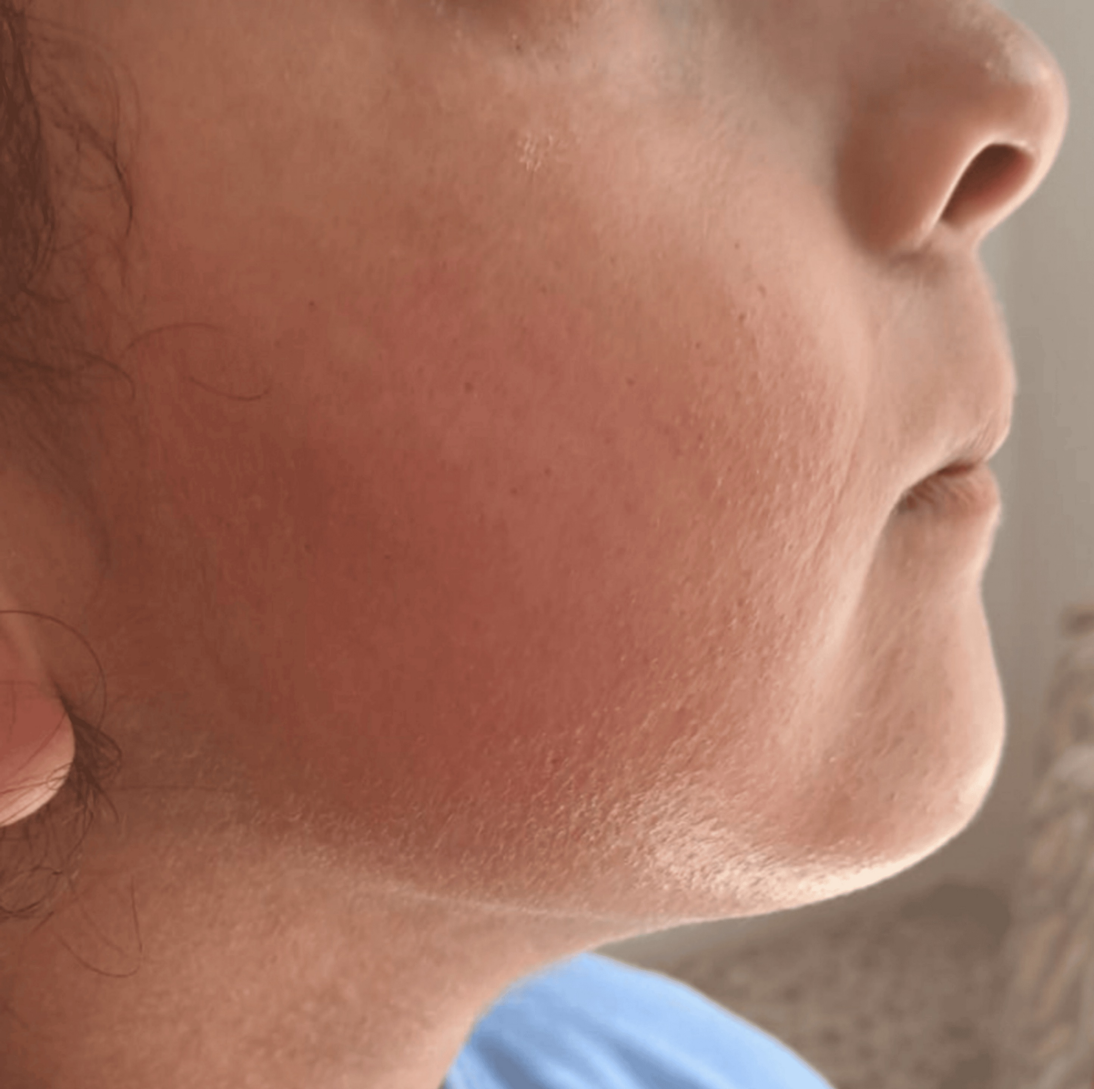
Hypersensitivity reaction with facial erythema occurring three hours after administration of articaine during a dental procedure in an 11-year-old girl

**Figure 2:**
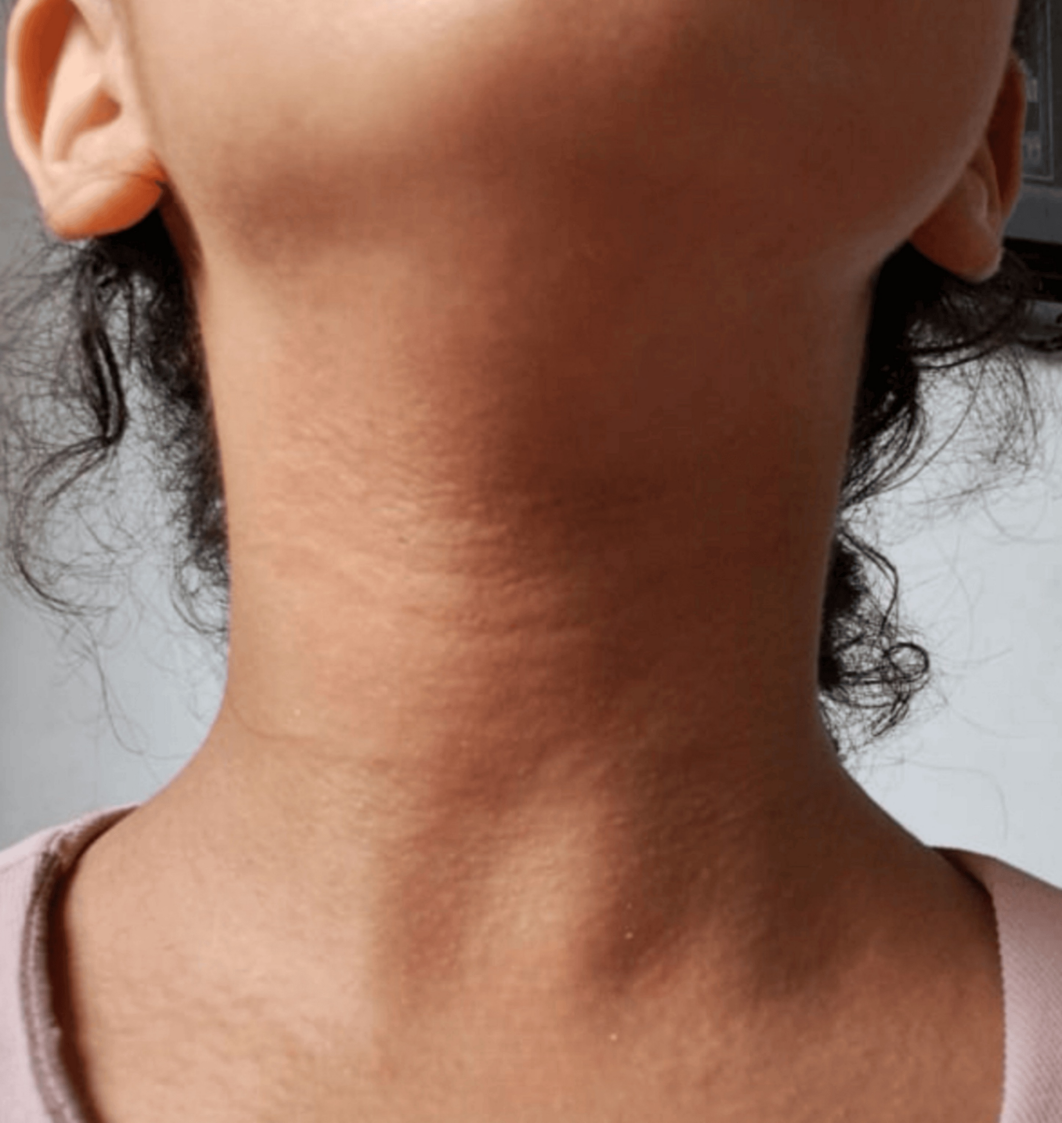
Hypersensitivity reaction presenting as erythema on the patient’s neck, observed three hours after administration of articaine during a dental procedure

An 11-year-old girl with a medical history of well-controlled asthma under maintenance therapy, no history of drug allergy, and no food intolerance, including sulfites, developed an erythematous and pruritic rash localized to the face and neck (Figure 1, Figure 2) approximately three hours after a dental procedure performed under articaine with epinephrine (40 mg/mL + 0.01 mg/mL). The eruption resolved spontaneously within a few hours.

The patient was referred to our department three months later for allergological evaluation prior to planned dental care. Her medical history revealed no prior exposure to medications, including antibiotics or nonsteroidal anti-inflammatory drugs, either before or after the procedure. Additionally, no topical benzocaine or other topical anesthetic was applied before the administration of articaine. Informed consent was obtained from the patient’s parents before initiating allergological investigations.

Skin testing was performed according to standard protocols. The positive control with histamine produced a 6-mm wheal, while the negative control with 0.9% saline was unremarkable. Skin prick tests (SPTs) and intradermal tests (IDTs) were conducted with LAs, including articaine + epinephrine, 2% lidocaine, mepivacaine, and bupivacaine. All SPTs with undiluted solutions remained negative after 15 minutes of observation. IDTs, starting at dilutions of 1/100 and subsequently 1/10, were also negative on immediate reading at 20 minutes. The latex SPT was negative. Given the relatively delayed onset of the initial reaction, delayed readings at 48 and 72 hours were additionally obtained, both of which remained negative.

**Figure 3:**
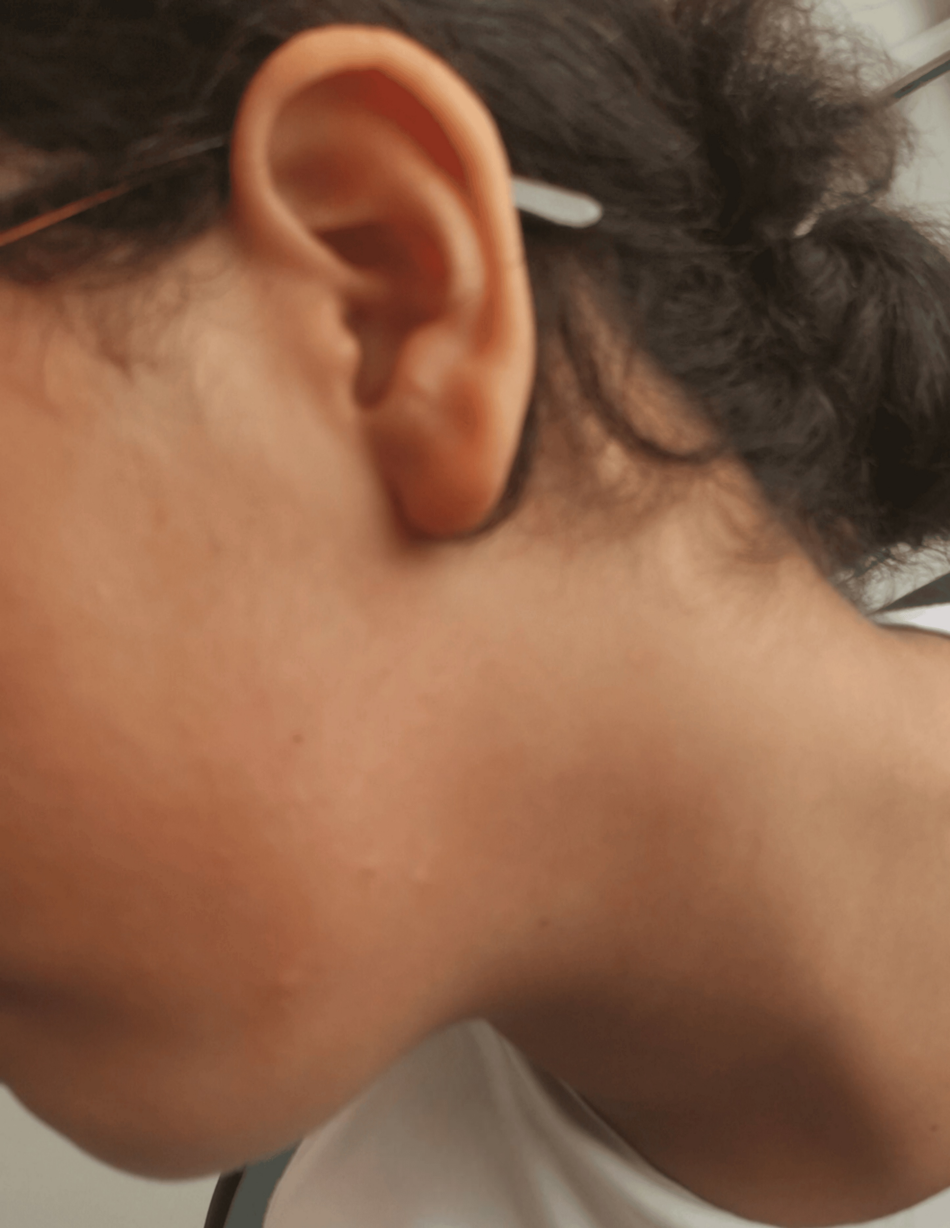
Hypersensitivity reaction presenting as pruritic erythema on the left cheek and neck, observed after subcutaneous administration of articaine during the drug provocation test in an 11-year-old girl

**Figure 4:**
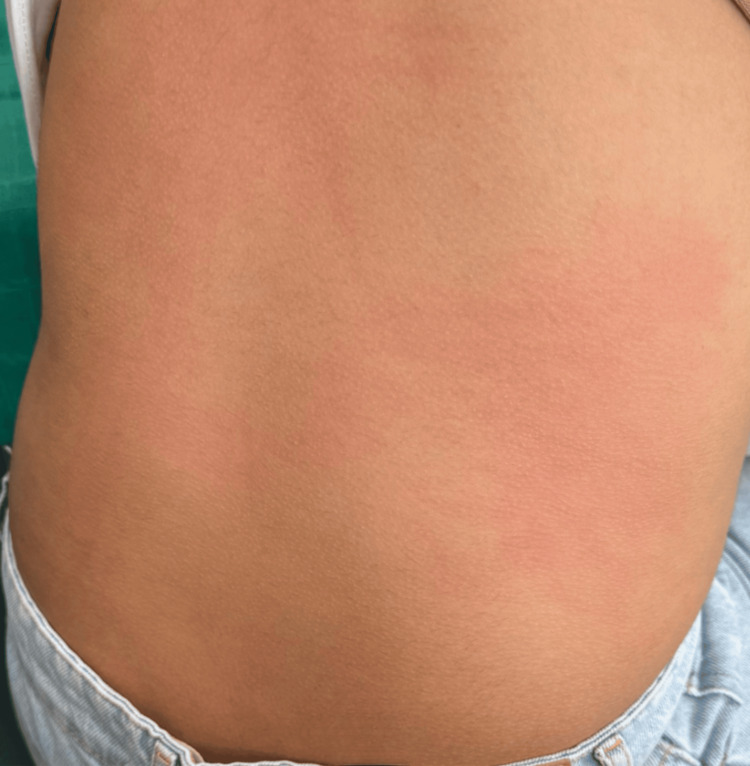
Hypersensitivity reaction presenting as pruritic erythema on the back, observed after subcutaneous administration of articaine during the drug provocation test in the patient

A drug provocation test with pure articaine was conducted in accordance with the European Network for Drug Allergy (ENDA)/European Academy of Allergy & Clinical Immunology (EAACI) recommendations. Subcutaneous injections of 0.1 mL, 0.5 mL, and 1 mL were administered at 30-minute intervals. Fifteen minutes after the third injection, the patient developed a hypersensitivity reaction with pruritus and erythema on the arms, back, and left cheek (Figure 3, Figure 4). She also developed acute wheezing and dyspnea associated with coughing. Pulmonary auscultation revealed bilateral sibilant rales, consistent with bronchospasm secondary to the LA. Management included oral desloratadine and continuous nebulized salbutamol for one hour, resulting in complete resolution. Serum tryptase measured 45 minutes after symptom onset was elevated at 23 µg/L (reference range: <11.4 µg/L), returning to 5 µg/L at 24 hours. The patient was discharged a few hours later after complete symptom resolution.

Two weeks after the initial evaluation, a new provocation test with mepivacaine without epinephrine was performed, yielding negative results after one day of clinical monitoring.

## Discussion

LAs are widely used in dental practice and are classified into two main groups: esters (e.g., procaine, benzocaine, and butacaine) and amides (e.g., articaine, lidocaine, and mepivacaine). Among these, articaine presents several distinctive characteristics [2]. Articaine is considered a hybrid LA molecule; it is an amide-type anesthetic with an additional ester group, allowing partial metabolism by plasma esterases and contributing to a shorter half-life. This pharmacological profile offers advantages such as faster onset, deeper tissue penetration, and efficient bone diffusion, making it one of the most widely used anesthetics in dental practice. LAs are generally well tolerated, and adverse reactions are uncommon [4]. Among these, hypersensitivity reactions may occur and can be either allergic or nonallergic. Nonallergic reactions are often due to direct mast cell degranulation, while allergic reactions may be IgE-mediated immediate-type (type I) or delayed-type (type IV) reactions driven by cell-mediated immunity without antibody release [5,6]. True allergic reactions to LAs are rare, estimated at around 1% of suspected cases [5], with esters more frequently implicated [3,7].

In a study of 331 patients conducted between 2000 and 2012, only one immediate allergic reaction and two delayed reactions were confirmed [8]. This low prevalence reflects the predominance of nonallergic mechanisms in most immediate reactions [6]. Excipients such as antioxidants and preservatives (e.g., methylparaben and metabisulfite) are well-known triggers of hypersensitivity [6], highlighting the need for caution in patients with documented food intolerance to sulfites (e.g., dried fruits, wine, vinegar, pickled products, and some processed or preserved foods). Historically, lidocaine was the most commonly used LA until 1976, after which articaine gained popularity, particularly in dentistry, due to its high efficacy even at low concentrations [2,5,9]. Articaine is considered among the best-tolerated LAs and is often proposed as a safe alternative in cases of suspected lidocaine intolerance [3,6].

In our setting, articaine remains the first-line anesthetic in dentistry. A cross-sectional survey of dental practitioners across multiple countries reported that 60% used articaine as their initial choice, often in combination with epinephrine to enhance efficacy through vasoconstriction and reduced bleeding. However, epinephrine may trigger vasovagal manifestations such as pallor, headache, or hypertension, which can be misinterpreted as allergic reactions. These manifestations likely account for a large proportion of the nonallergic hypersensitivity reactions described above [3,4]. Although rare, confirmed cases of articaine allergy have been reported, but the true prevalence remains uncertain [4]. In children, LAs are used not only in dental procedures but also in minor surgical interventions such as circumcision [1,7].

Pediatric studies investigating LA allergy are scarce, but the incidence appears comparable to that in adults [5,7]. Clinical manifestations of type I hypersensitivity include erythema, pruritus (as observed in our patient: Figure 1, Figure 2), urticaria, angioedema, bronchospasm, and anaphylaxis [4,5]. In our patient, the immediate hypersensitivity during the first dental procedure prompted an allergy workup to confirm the implication of articaine and to identify a safe alternative for subsequent dental care. The allergy evaluation aimed to establish an IgE-mediated mechanism and followed the ENDA/EAACI recommendations [10], whereby children with a history of hypersensitivity to LAs undergo testing. Notably, comorbidities such as asthma, food allergy, or hypersensitivity to other drugs are not, by themselves, indications for LA testing [11]. In our case, despite the presence of asthma, the reported reaction to articaine justified further allergological evaluation.

Evaluation of immediate hypersensitivity relies on skin testing, ideally using preservative- and epinephrine-free formulations, though these are not always available in clinical practice [3,4]. Testing is recommended at least four weeks after the initial reaction to reduce false positives [7]. SPT is performed with undiluted solutions, and results are read after 15 minutes using appropriate positive and negative controls. If negative, IDT with up to a 1/10 dilution of the pure solution may be performed [10]. Both tests have comparable diagnostic value [4] and may confirm IgE-mediated allergy if positive [11].

Drug provocation testing remains the gold standard for diagnosis, with a high negative predictive value and the added benefit of identifying safe alternatives [1,12]. This test is indicated when intradermal results are negative and involves graded administration of three doses (0.1, 0.5, and 1 mL) of the pure LA solution at 30-minute intervals [13]. In our patient, drug provocation with articaine reproduced the allergic reaction, with erythema, pruritus (Figure 3, Figure 4), and bronchospasm after the third dose, confirming an IgE-mediated mechanism. A subsequent negative provocation test with mepivacaine identified it as a safe alternative, consistent with previous literature [11]. Cross-reactivity between esters and amides is rare, although cases of cross-reactivity within amides, including articaine, have been reported [4,6].

It is also essential to investigate other agents potentially encountered during dental procedures, such as latex [13], which was excluded in our patient with a negative SPT. Laboratory testing is of limited value due to the lack of specific IgE assays for LAs. In our case, an elevated serum tryptase supported the anaphylactic nature of the systemic reaction, helping to rule out vasovagal syncope or direct nonimmune adrenergic effects, while comparison with a normal baseline level excluded underlying mast cell disorders, particularly in the absence of severe or recurrent reactions [4]. Where allergy evaluation is not feasible or fails to identify a safe alternative, diphenhydramine or general anesthesia may be considered as measures of last resort and are relatively safe [6].

The main limitation of this report lies in the use of articaine preparations containing epinephrine and sodium metabisulfite, as preservative-free formulations were unavailable. Nevertheless, the allergy workup strongly supports an IgE-mediated reaction to articaine.

This case contributes to the limited literature and aims to raise awareness among dental professionals about the potential for allergic reactions to LAs and their clinical presentations. It also underscores the need for continuous staff training in the early recognition and management of acute allergic reactions. Regular simulation-based training and emergency preparedness protocols should be implemented in dental and medical settings to ensure timely treatment of anaphylaxis or bronchospasm. Finally, maintaining readily available emergency medications in dental offices is essential for prompt and effective management.

## Conclusions

Articaine, one of the most commonly used LAs in dental practice, is rarely associated with confirmed allergic reactions. This case contributes to the limited literature and emphasizes the importance of recognizing hypersensitivity reactions, acquiring appropriate management skills, and ensuring the availability of emergency treatments in dental settings. Given the rarity of confirmed cases, the risk-benefit profile continues to favor the use of articaine, which is effective and generally well tolerated. Safe alternatives exist when allergy is confirmed, highlighting the central role of allergological evaluation in diagnosis.

## References

[1] Caliskan N, Yildirim G, Bologur H (2024). Local anesthetics allergy in children: evaluation of diagnostic tests with real-life data. Pediatr Allergy Immunol.

[2] Li L, Sun DL (2023). Adverse effects of articaine versus lidocaine in pediatric dentistry: a meta-analysis. J Clin Pediatr Dent.

[3] Al-Dosary K, Al-Qahtani A, Alangari A (2014). Anaphylaxis to lidocaine with tolerance to articaine in a 12 year old girl. Saudi Pharm J.

[4] Halling F, Neff A, Meisgeier A (2025). True allergies to articaine: a 25-year analysis. Dent J (Basel).

[5] Aslan S, Anıl H, Kaya M, Harmancı K (2025). Evaluation of diagnostic tests for immediate-type allergic reactions to amide group local anesthetics in children. Pediatr Allergy Immunol.

[6] Dey M, Mishra BP, Awasthi D, Sahoo A (2020). Articaine as an alternative in lidocaine allergy: case report of a seventy year old male patient. Int J Surg Case Rep.

[7] Selmanoglu A, Güvenir H, Celik IK, Karaatmaca B, Toyran M, Civelek E, Misirlioglu ED (2021). Immediate local anesthetic reactions and diagnostic test results in pediatric patients. Allergol Immunopathol (Madr).

[8] Batinac T, Sotošek Tokmadžić V, Peharda V, Brajac I (2013). Adverse reactions and alleged allergy to local anesthetics: analysis of 331 patients. J Dermatol.

[9] Shree R, Kedia MR, Toshi T, Raj N, Anand K, Shahi N (2022). A cross-sectional study on the evidence-based dentistry, perception basis, and use of articaine among dental practitioners. Cureus.

[10] Demoly P, Adkinson NF, Brockow K (2014). International Consensus on drug allergy. Allergy.

[11] YIlmaz I, Özdemir SK, Aydin Ö, Çelik GE (2018). Local anesthetics allergy: who should be tested?. Eur Ann Allergy Clin Immunol.

[12] Trautmann A, Goebeler M, Stoevesandt J (2018). Twenty years' experience with anaphylaxis-like reactions to local anesthetics: genuine allergy is rare. J Allergy Clin Immunol Pract.

[13] Broyles AD, Banerji A, Barmettler S (2020). Practical guidance for the evaluation and management of drug hypersensitivity: specific drugs. J Allergy Clin Immunol Pract.

